# Treatment of Failed Patella ORIF With Novel Patella Tension Band Plate and Suture Construct: A Case Report

**DOI:** 10.1155/cro/6150955

**Published:** 2026-07-11

**Authors:** Hrayr Basmajian, Cailan L. Feingold, Eric H. Lin, Ryan R. Mayer, Christopher B. Hayes, Nirav H. Amin, Joseph N. Liu

**Affiliations:** ^1^ Department of Orthopaedic Surgery, Premier Orthopaedic and Trauma Specialists, Pomona, California, USA; ^2^ Department of Orthopaedic Surgery, Keck School of Medicine of USC, Los Angeles, California, USA, usc.edu

**Keywords:** case report, comminuted, inferior pole patella fracture, patella fracture, patella plate, revision patella fracture

## Abstract

Hardware failure and malunion are feared complications of open reduction and internal fixation (ORIF) of patella fractures and require revision ORIF. Given the higher complication and reoperation rates associated with revision ORIF of the patella, these procedures can be difficult. This case report presents a 51‐year‐old female who experienced early hardware failure and malunion of a patellar fracture after primary ORIF with tension band wiring (TBW). A revision ORIF was performed using a novel plate and suture construct that allows for the simultaneous management of the osseous and soft tissue components of the extensor mechanism.

## 1. Introduction

Fractures of the patella are common, accounting for about 1% of skeletal injuries [1]. Patellar fractures can be managed both conservatively and operatively, depending on the characteristics of the injury and the patient [1, 2]. Fractures that are displaced by more than 1–4 mm, fractures disrupting the extensor mechanism, and comminuted fractures are all injuries that warrant operative management [3, 4]. Restoring the structure of the patella is crucial as it plays a key role in the extensor mechanism, facilitating knee extension, centralizing the force of the quadriceps muscles, and protecting the anterior aspect of the knee [5, 6].

Surgical management of patellar fractures includes open reduction and internal fixation (ORIF) with the use of tension bands with Kirschner wires or cannulated screws or lag screws [4]. Rates of fixation failure following ORIF have been reported to be as high as 11%, and it is challenging to manage with revision procedures being associated with high complication rates [7–10]. Additionally, when they fail, it is usually early in the postoperative course, complicating matters further with acute surgical wounds. In this case report, we present a patient with failed fixation following ORIF who successfully underwent revision ORIF with a novel patella plating system.

## 2. Case Presentation

**Figure 1:**
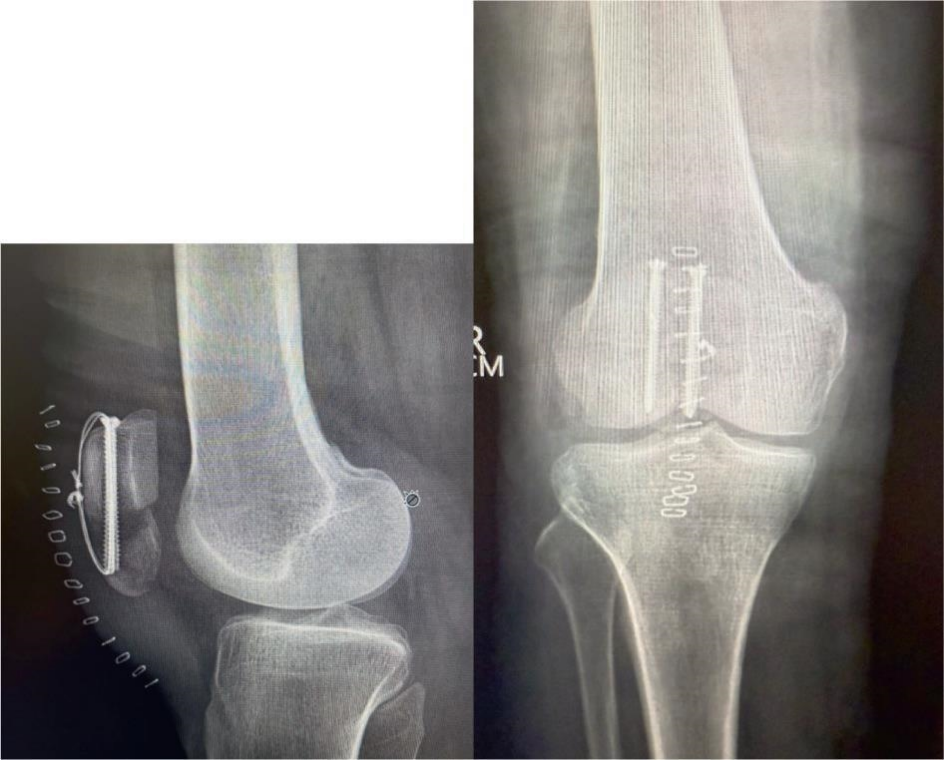
Preoperative lateral and AP radiographs demonstrating hardware failure due to pullout from the inferior pole fragment.

The patient is a 51‐year‐old female who suffered an injury to her right patella 18 days prior. One day after the initial injury, the patient underwent ORIF with cannulated screws, tension band wiring, and repair of the medial and lateral retinaculum. Upon arrival at our clinic, radiographs demonstrated hardware failure with signs of loosening. Radiographs also showed that the hardware had pulled through the inferior comminuted fragment (likely secondary to fracture fragment displacement), creating suboptimal potential for healing (Figure 1). Physical exam of the right lower extremity demonstrated a wound with staples that was clean, dry, and intact, mild swelling, inability to maintain a straight leg raise, neurovascularly intact distally, and palpable dorsalis pedis pulse.

**Figure 2:**
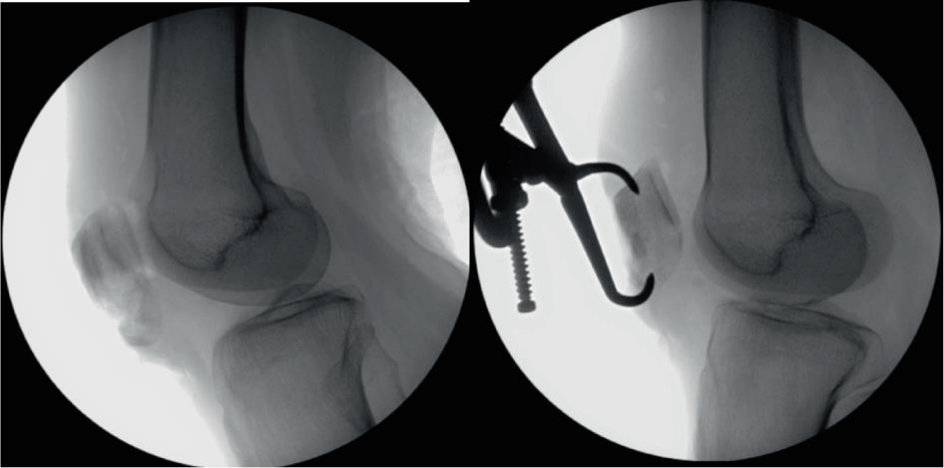
Fluoroscopic lateral images with confirmation of patellar fracture reduction using point‐to‐point clamps prior to revision fixation.

Eighteen days following the initial surgery, the patient was brought to the operating room for the removal of the original hardware and revision ORIF. Following careful removal of the original hardware and debridement at the fracture site, point‐to‐point clamps were used along with AP and lateral imaging using fluoroscopy to confirm appropriate fracture reduction (Figure 2).

**Figure 3:**
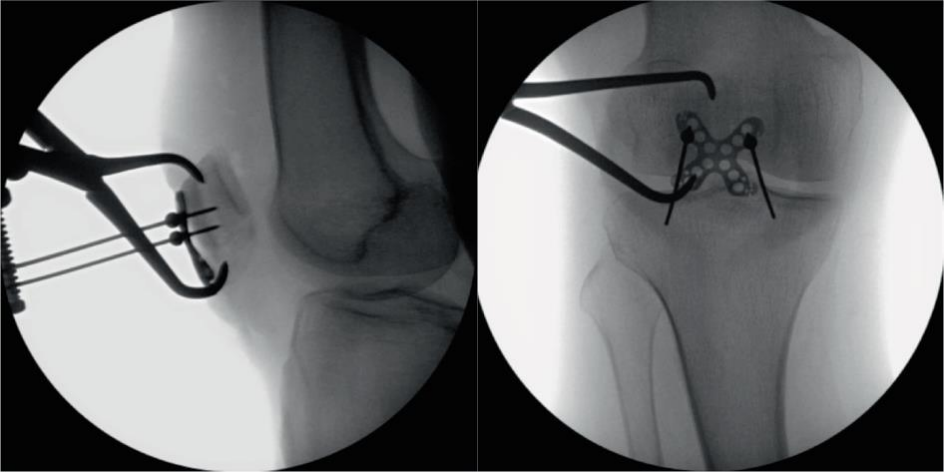
Fluoroscopic AP and lateral images confirming plate position and fracture reduction prior to final fixation of patella‐specific plate with integrated sutures.

The Summit Patella Plate (Endeavor Orthopaedics, Tulsa, OK) was then placed anteriorly over the patella to stabilize the fracture. Ilizarov Olive Wires were used to stabilize the plate before final fixation to confirm appropriate fracture reduction (Figure 3).

**Figure 4:**
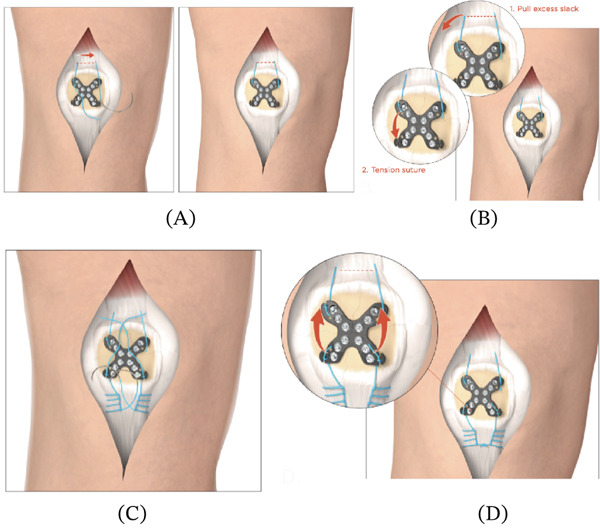
(A) Superior tension band suture placement diagram. (B) Illustration demonstrating the mechanism to adjust tension on the sutures. (C) Patellar tendon Krakow suture technique. (D) Patellar tendon suture tensioning. *Source:* Endeavor Orthopaedics.

After confirmation of plate position, the plate was fixed using eight locking screws, two in each of the four arms of the plate. The single superior preloaded #2 suture was then used to create a tension band construct through the quadriceps tendon. This suture, which was preloaded on the patella plate, was passed approximately 3 cm above the quadriceps insertion on the patella from lateral to medial and back down to the other superior arm of the plate; the suture eyelet behind the needle was used to attach to the superior arm by sliding over the cleat (Figure 4). For this plate′s design, the suture is always passed from left to right. The suture was then tensioned by pulling the excess slack back from the original superior arm with care not to overtighten the suture. Flexing the knee to 30° assisted in achieving appropriate tension, and some slack on the suture while in full extension was acceptable. After achieving the desired tension, the suture was cut from the original superior arm, leaving a 10‐mm tail.

**Figure 5:**
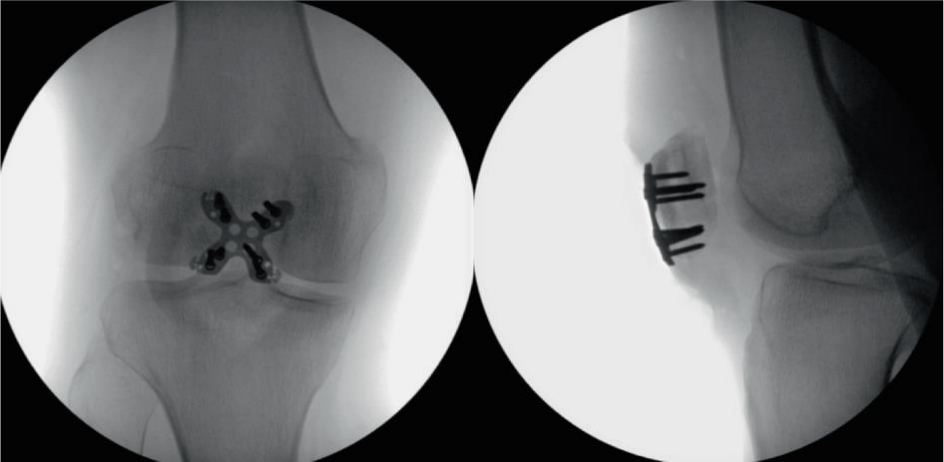
Nonstress fluoroscopic AP and lateral images demonstrating the final fixation plate with eight screws.

Using the two preloaded sutures from the two inferior arms of the plate, a Krakow suture technique was performed in the patellar tendon (Figure 4). After performing the Krakow sutures, the needles were taken to the center of the tendon, and a long enough tail was left bilaterally to allow the two suture tails to be tied together. Slack in the Krakow sutures was pulled superiorly from the plate to tension the construct. Tensioning was again performed with the knee in 30° of flexion. Once the desired tension was achieved and confirmed after taking the knee through a full range of motion, the sutures were cut from the plate, leaving a 10‐mm tail. Nonstress fluoroscopic images were then taken to confirm adequate fracture reduction and fixation (Figure 5).

**Figure 6:**
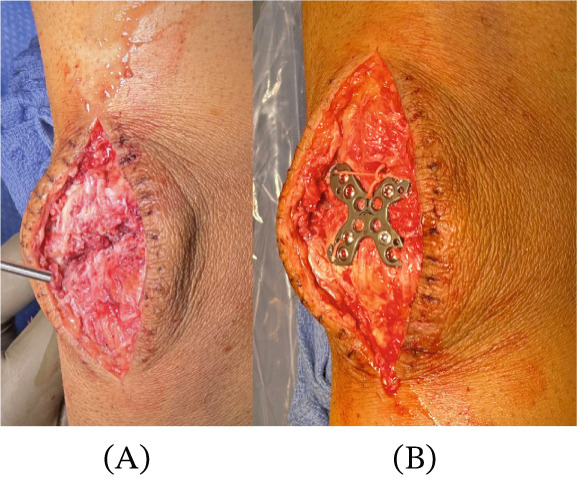
Photos demonstrating (A) the patella following hardware removal and (B) after revision ORIF with novel plate and suture construct.

The operative site was irrigated with saline and then closed with 0 Vicryl and 2‐0 Vicryl sutures, and a negative pressure wound vacuum‐assisted closure was placed. Postoperatively, the patient was made weight‐bearing as tolerated, locked in extension for 6 weeks. Table 1 details the postoperative protocol. Figure 6 demonstrates intraoperative images at the start and end of the surgical procedure.

**Table 1:** Postoperative rehabilitation protocol followed. Key: Weight‐bearing as tolerated (WBAT).

Time postoperative	Rehabilitation guidelines
Weeks 0–4	WBAT; locked in extension
Weeks 4–6	WBAT locked in extension; brace unlocked to 30° flexion during rest
Week 6	Brace unlocked for ambulation; brace unlocked for an additional 30° flexion; initiate quadriceps strengthening; weight‐bearing closed chain strengthening initiated
Week 9	Loaded knee extension initiated

**Figure 7:**
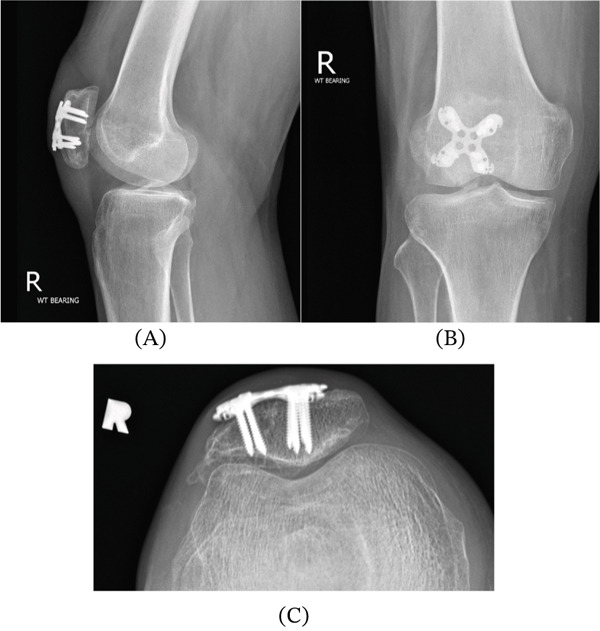
Radiographs demonstrating (A) lateral, (B) AP, and (C) sunrise views at 5 months postoperative with healed fracture and intact hardware.

**Figure 8:**
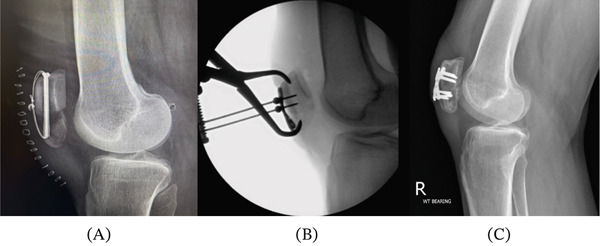
Lateral images demonstrating (A) failure of original fixation, (B) intraoperative reduction, and (C) final postoperative images illustrating union at 5 months postoperative.

The patient returned to work on modified duties at 5 months postoperatively (Table 2). At 7 months postoperative, the patient′s range of motion was 0°–130°, with quadriceps strength improving (Table 1). Incision remained clean, dry, and intact throughout the postoperative period. Figure 7 contains radiographs demonstrating the healed fracture with the hardware in place at 5 months postoperative. Figure 8 compares lateral imaging of the failure of the original construct, intraoperative reduction, and union at 5 months postrevision. At 11 months postoperative, the patient′s Lysholm score was 79/100 and Kujala score was 69/100.

**Table 2:** Timeline table.

Timepoint	Event
Day 0	Original patella injury sustained
Day 1	Primary ORIF with TBW construct
Day 19	Hardware removal and revision ORIF with novel plate construct
5 months	Plain radiographs demonstrate healing fracture with intact hardware, returned to work on modified duties
7 months	ROM 0‐130 Quadriceps strength improving

## 3. Discussion

Management of failed ORIF of patellar fractures can be difficult and carries a higher risk of complications. This case report presents the use of a novel plate and suture construct for revision ORIF of the patella. This fixation system presents an optimal way to manage both patella fractures and the associated soft tissues, as the ability to tension both the quadriceps and patellar tendon allows the surgeon to neutralize the forces of the quadriceps and patella to directly load the plate rather than the fracture.

This patient experienced an early failure of the initial construct that was likely multifactorial in nature, related to both technical and biological factors. For example, the literature demonstrates that fractures to the inferior pole of the patella exhibit significantly more comminution [11, 12]. Comminution was identified in this patient′s fracture pattern on their first visit to our clinic and may have been unappreciated on initial radiographs. Lazaro et al. explain that CT demonstrated severely comminuted distal pole fractures identified in 88% of cases but were unappreciated on plain radiographs in 44% of those cases [12]. Preoperative CT may have further elucidated the fracture pattern before initial surgery. Although TBW is effective in simple transverse fractures, it is ineffective in the presence of comminution [13]. Identification of comminution on preoperative imaging may have changed the planned fixation type, given that TBW can be associated with reduction loss and fixation failure in comminuted distal pole fractures. This is because the distal pole has limited cortical bone to help achieve adequate purchase for TBW constructs [11]. Isolated plating using anterior‐to‐posterior direct screws is a construct that still relies on bone quality, which is suboptimal for fixation in the inferior pole in the setting of comminution [10]. Therefore, the bone quality of the distal pole, as well as the presence of comminution, made this fracture difficult to fix with either TBW or plating in the initial surgery, and these factors likely contributed to the initial failure of the TBW.

Other factors that can influence loss of fixation include patient‐related factors. Although we do not know if these played a role in this patient′s loss of fixation, they are well‐documented within the literature. Noncompliance with postoperative restrictions is associated with failure of TBW in patella fractures [9, 14]. However, other studies have found that patient‐specific factors were not found to be associated with increased complications [15]. Patient education on postoperative activity restrictions is important, nonetheless, to help achieve optimal outcomes.

For this patient′s revision ORIF, plating alone would be less effective as it still relies on the inferior pole bone quality. The construct utilized in this patient reflects a new surgical strategy that reduces the reliance on fixation on patellar bone quality, which in this case is diminished due to inferior pole comminution. This construct does this by neutralizing the forces of the quadriceps tendon and patellar tendon using the suture technique and transmission of the force of the quadriceps tendon to the plate and then to the patellar tendon directly.

Postoperative function of the extensor mechanism is an important concern of the surgeon addressing patellar fractures surgically. Extensor mechanism insufficiency is a potential complication of injuries to the patella and operative fixation of patella fractures [16]. Using a construct like this patella‐specific plate with integrated sutures, which addresses both the osseous injury as well as neutralizing the constant pull of the patellar and quadriceps tendons, may help to improve extensor mechanism functionality postoperatively. This is especially valuable for patients undergoing revision procedures, who are at a higher risk for poor outcomes and complications [17].

Use of a plate for ORIF of the patella may also offer an advantage over standard tension band wiring (TBW) techniques, which had previously failed for this patient. ORIF with plating for patellar fractures is a safe alternative to TBW and may be associated with fewer complications and reoperations [18, 19]. Other research has shown favorable clinical outcomes and confirmed radiological union by 3 months postoperatively in all patients who underwent ORIF with a plate [20]. Biomechanical studies also prove superior construct stability in plates than TBW [21, 22]. Given the potentially lower complication rates and improved construct stability supplied by plating of patella fractures, they may be the preferred technique for patella ORIFs.

For revision procedures for inferior pole patella fractures, this plating construct addresses the shortcomings of other non‐TBW alternative constructs. Partial patellectomy is an alternative option for revision; however, it is associated with poorer outcomes and patella baja compared to bone‐preserving techniques [23]. Suture anchor fixations are another alternative to TBW and plating that are effective for inferior pole fractures [24]. However, a concern for suture anchor fixations in the revision setting would be anchor pullout in compromised bone quality. Biomechanical studies have demonstrated decreased performance of suture anchors in osteoporotic bone, which may compromise their performance in bone that has been degraded by prior surgery and failed fixation [25]. Basket plate osteosynthesis has been shown to work well for inferior pole fractures and preserve patellar height [23]. Basket plates also require adequate screw purchase, which could be compromised in this patient′s case. The plate utilized in this technique, though, reduces reliance on bone quality and simultaneously addresses both osseous and soft tissues, making it an ideal construct for this patient′s revision.

Hardware irritation following ORIF of the patella is a potential concern, given the limited soft tissue superficial to the bone. Checketts et al. demonstrated a very low risk of hardware removal (5%–7%) with dorsal plating using 2.0‐ and 2.4‐mm‐thick plates [19]. The size of this plate (standard size: length 30.5 mm, width 29.1 mm, and thickness 1.25 mm) is even thinner than the plates reported by Checketts et al., making the need for hardware removal likely lower [19]. Greenberg et al. demonstrated that removal of hardware at a mean of 15.8 months following patellar fracture fixation improved patient‐reported pain and quality of life [26]. This plate can be easily removed if necessary by removing the screws, cutting the sutures off each arm, lifting the plate from the patella, and removing any suture remnants from the soft tissues. If this patient were to develop hardware irritation down the road in her postoperative course, the plate could therefore be removed once the fracture has healed.

An important limitation to acknowledge in the findings in this case report is the absence of preoperative patient‐reported outcome measure scores to compare to the postoperative scores. Future studies or case reports that use this construct can improve our understanding of its success by incorporating standardized patient‐reported outcome measures and comparing them preoperatively and postoperatively.

## 4. Conclusion

This case report demonstrates the successful use of a novel plate and suture construct to successfully manage a malunion of the patella following hardware failure. It carries the potential advantage of addressing both osseous and soft tissue elements of the extensor mechanism, potentially helping to protect the biomechanics of knee extension. Future comparative and biomechanical studies may evaluate the efficacy of this patella‐specific plate with integrated sutures construct in terms of time to return to activity, pain scores, and patient functional outcomes compared with other ORIF techniques.

## Funding

No funding was received for this manuscript.

## Disclosure

The manufacturer of the plate used in this study did not influence study design, analysis, or writing.

## Ethics Statement

Informed consent was obtained from the patient for publication. This protocol was approved by the University of Southern California′s IRB HS‐25‐00098.

## Conflicts of Interest

Hrayr Basmajian MD reports a relationship with Acumed LLC that includes paid consultant, Smith and Nephew that includes paid consultant, Stryker that includes paid consultant, and ITS that includes design team. Christopher B. Hayes MD reports a relationship with Decision Support in Medicine LLC that includes publishing royalties and financial or material support. Joseph N. Liu reports a relationship with Stryker Orthopaedics that includes speaking and lecture fees and Innocoll Biotherapeutics NA Inc that includes travel reimbursement. Nirav H. Amin MD reports a relationship with CONMED Corporation (type: other professional activities), Zimmer Biomet Holdings, Inc. (type: other professional activities), Smith and Nephew (type: other professional activities), ABANZA (type: stock), and OSSIO Inc (type: other professional activities. Cailan L. Feingold, Eric H. Lin, and Ryan R. Mayer MD declare no conflicts of interest.

## Data Availability

Data are available from the corresponding author upon reasonable request.
